# Multifaceted Biological Activity of Rutin, Quercetin, and Quercetin’s Glucosides

**DOI:** 10.3390/molecules30122555

**Published:** 2025-06-11

**Authors:** Danuta Zielińska, Małgorzata Starowicz, Małgorzata Wronkowska, Henryk Zieliński

**Affiliations:** 1 Department of Chemistry, University of Warmia and Mazury in Olsztyn, Plac Łódzki 4, 10-721 Olsztyn, Poland; danuta.zielinska@uwm.edu.pl; 2 Team of Chemistry and Biodynamics of Food, Institute of Animal Reproduction and Food Research, Polish Academy of Sciences, Trylińskiego 18, 10-683 Olsztyn, Poland; m.starowicz@pan.olsztyn.pl (M.S.); m.wronkowska@pan.olsztyn.pl (M.W.)

**Keywords:** rutin, quercetin, quercetin glucosides, antioxidant potency, anti-glycation, angiotensin-converting enzyme inhibition, acetylcholinesterase inhibition

## Abstract

In this study, the ranking of the multifaceted activity of rutin (Ru), quercetin (Q), and quercetin’s glucosides (Q3G, Q4′G and Q3,4′G) was addressed. The antioxidant potency was determined by electrochemical methods, whereas the ability of these compounds to inhibit angiotensin-converting enzyme (ACE) activity, acetylcholinesterase (AChE) activity, and advanced glycation endproduct (AGE) formation was examined in bovine serum albumin (BSA)/glucose and BSA/methylglyoxal (MGO) model systems to show their importance against hypertension, Alzheimer-type dementia, and diabetic complication, respectively. Then, the relationship between the biological activities of these compounds and their antioxidant potential provided by the cyclic voltammetry (CV) method was evaluated. The ranking of the ACE inhibitory activity was Q > Q3,4′G > Ru > Q3G > Q4′G. The correlation coefficient between ACE enzyme inhibitory activities and antioxidant potentials had a value of r = −0.68, thus clearly indicating the impact of antioxidant potential and chemical structure on ACE inhibitory activity. The ranking of the AChE enzyme inhibitory activity was Q ≈ Q3G ≈ Q4′G ≈ Ru > Q3,4′G, and the correlation between their antioxidant potentials and AChE inhibitory activities (r = −0.77) also indicated the impact of chemical structure. The quercetin glucosides displayed strong inhibitory capacity on AGE formation, as the ranking of anti-AGE activity in the BSA/MGO model system was Q3,4′G ≈ Q4′G ≈ Q3G > Ru ≈ Q > AG. The anti-AGE activity of rutin, quercetin, and quercetin’s glucosides was negatively correlated with their IC_50_ values for ACE inhibition (r = −0.67) and AChE inhibition (r = −0.81), whereas no correlation was found between their ACE and AChE inhibition activities. These effects of rutin, quercetin, and quercetin’s glucosides expand our knowledge of the multifunctional activity of biologically active compounds of plant origin.

## 1. Introduction

Food of plant origin contains phytochemicals that are of considerable interest in the prevention or delaying of the development of degenerative diseases [1,2]. In this context, the multifunctional activity of flavonols such as rutin, quercetin, and their glucosides has attracted attention due to their naturally high content in fruits and vegetables, especially in fresh and stored onions, where they are found mainly in glucoside and aglycone forms [3,4]. Fossen et al. [5] and Zielinska et al. [4] showed that quercetin in onions exists in four predominant forms: quercetin aglycone, quercetin-3,4′-di-*O*-β-glucoside, quercetin-3-*O*-β-glucoside, and quercetin-4′-*O*-β-glucoside, whereas rutin (quercetin-3-*O*-rutinoside) is usually considered alongside these compounds due to its similarity in chemical structure. Various model systems have been applied to the characterization of the multifunctional activity of biologically active compounds. In this paper, the term “multifunctional activity” is related to these compounds’ antioxidant activity, inhibition of angiotensin-converting enzyme activity, inhibition of acetylcholinesterase activity, and inhibition of advanced glycation end-product formation.

Different protocols for the measurement of the antioxidant potential of phytochemicals have been reported [6]. Traditional antioxidant assays (e.g., DPPH radical scavenging, ABTS decolorization, FRAP, ORAC) are widely used but have limitations such as extensive sample preparation, the use of hazardous reagents, long reaction times, and sometimes poor selectivity. Moreover, spectrophotometric assays often cannot distinguish individual redox-active compounds. Among them, electrochemical methods such as cyclic (CV) and staircase voltammetry (SCV) for antioxidant assays have emerged in the past decade [7,8,9]. In this study, we applied cyclic voltammetry (CV) as this technique has gained prominence for evaluating antioxidant content in complex food matrices as well as the electron-donating capacity of molecules. CV involves sweeping the electrode potential and recording the resulting current, generating a voltammogram that reflects the presence and redox behavior of antioxidants. The voltammogram thus provides qualitative information (oxidation peak potentials indicating antioxidant strength) and quantitative information (peak currents or charges proportional to antioxidant concentration). CV requires minimal sample preparation—often an aqueous or hydroalcoholic extract/standard solution is mixed with supporting electrolyte and analyzed. The technique is fast (a scan takes seconds) and can provide valuable information on the electroactivity of samples or compounds in one run if the peaks are distinguishable [8,9,10,11,12,13,14]. A distinguishing feature of CV is that it senses only redox-active substances—for food antioxidants, these typically include phenolic compounds (flavonoids, phenolic acids, etc.), ascorbic acid, some vitamins, and Maillard reaction products with reducing power. There is scarce information on the relationship between electroactive molecules and their inhibitory activity against ACE activity, AChE activity, and advanced glycation endproduct (AGE) formation.

Hypertension or high blood pressure affects one-quarter of the world’s adult population, and it is one of the most important risk factors that contributes to the development of cardiovascular disease (CVD) [15,16]. The regulation of blood pressure involves pharmacological therapies, including angiotensin-converting enzyme (ACE) inhibitors [17,18] and non-pharmacological management related to lifestyle changes and a healthy diet [16]. Angiotensin-I-converting enzyme (ACE, EC 3.4.15.1) plays a key physiological role in the control of blood pressure [19,20,21]. Synthetic drugs that inhibit ACE, such as captopril and enalapril, can have negative side effects [21]. Recently, scientific studies have shown the positive impact of food with a potent ACE inhibitory activity, such as casein [22], tuna bone protein [23], soybean [24], wheat germ [25], green tea [26], and many others [27,28,29]. At present, scarce information is available regarding the inhibitory activity of quercetin and its glucosides against angiotensin-I converting enzymes [30,31].

From the basic research perspective, evaluation of the ACE inhibitory activities and antioxidant/reducing activity of quercetin and its glucosides may provide new knowledge in the field of functional properties verification of these compounds.

Neurological disorders, especially Alzheimer’s disease (AD), are characterized clinically within the elderly population by a loss of cognitive ability, severe behavioral abnormalities, and ultimately death [32,33,34]. A deficiency in levels of the neurotransmitter acetylcholine (ACh) has been observed in the brains of AD patients. Therefore, one of the strategies applied for the treatment of Alzheimer’s dementia to enhance cholinergic functions is the use of selective (AChE) inhibitors to increase the amount of ACh present in the synapses between cholinergic neurons [32,35]. The natural phytochemicals identified in plants seem to be good options for AChE inhibitors, and alkaloids, flavonoids—together with flavonols—and phenols represent important and interesting classes of biologically active inhibitors [36,37].

Multifaceted biological activity also covers the inhibition of advanced glycation end-product (AGE) formation. AGEs have been proposed to be causative factors for various kinds of diseases, especially diabetes [38,39]. They are the most important proinflammatory compounds that originate from the Maillard reaction, which is a fundamental reaction occurring in thermally treated food that has nutritional and toxicological effects on processed food. There is a basic difference between dietary AGEs from the Maillard reaction and those generated endogenously in human organisms. The sum of all possible adducts formed from sugars and proteins, independent of their structure, constitute endogenous AGEs. The formation of AGEs is correlated with aging and with the development of different diseases like diabetes, uremia, cataracts, atherosclerosis, or Alzheimer’s disease [40]. Diabetes is a high-prevalence major chronic metabolic disease demanding effective interventions, and the application of the natural flavonoid quercetin for the treatment of diabetes has recently been reported [41,42,43]. The major mechanism of the anti-glycation activity mediation caused by various phenolic antioxidants from plant extracts results from their ability to inhibit free radical generation in the glycation process [28,44,45,46].

**Figure 1:**
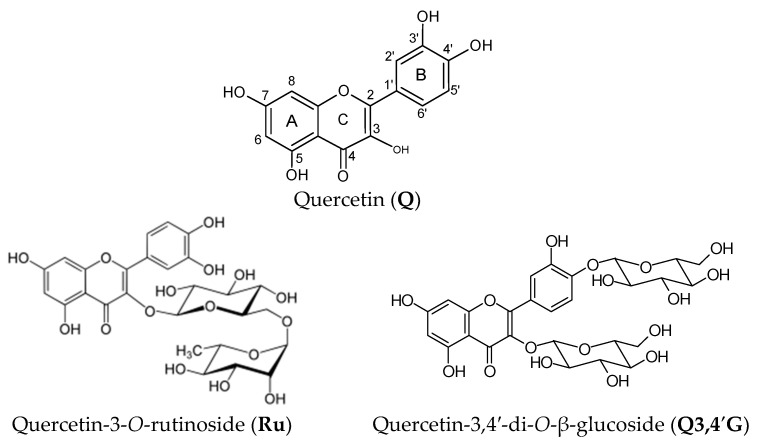

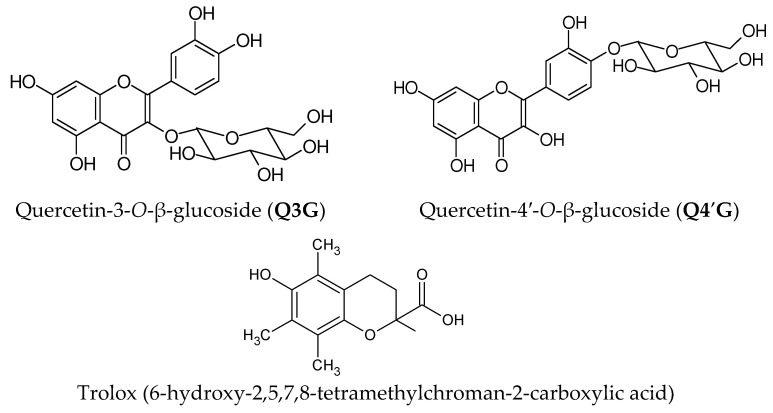
The structures of the compounds under investigation.

The aim of this study was to demonstrate the multifaceted activity of rutin, quercetin, and quercetin’s glucosides (Figure 1). Antioxidant potency was determined by electrochemical methods, whereas the ability of these compounds to inhibit angiotensin-converting enzyme activity, acetylcholinesterase activity, and the non-enzymatic glycation of proteins was performed in model systems to show their importance against hypertension, Alzheimer-type dementia, and diabetic complications, respectively. Then, the relationships between the multifaceted activity of these compounds and their antioxidant potentials, provided by the cyclic voltammetry (CV), method were evaluated.

## 2. Results and Discussion

### 2.1. The Antioxidant Potency of Rutin, Quercetin, and Quercetin’s Glucosides Provided by the Voltammetric Experiments

**Figure 2:**
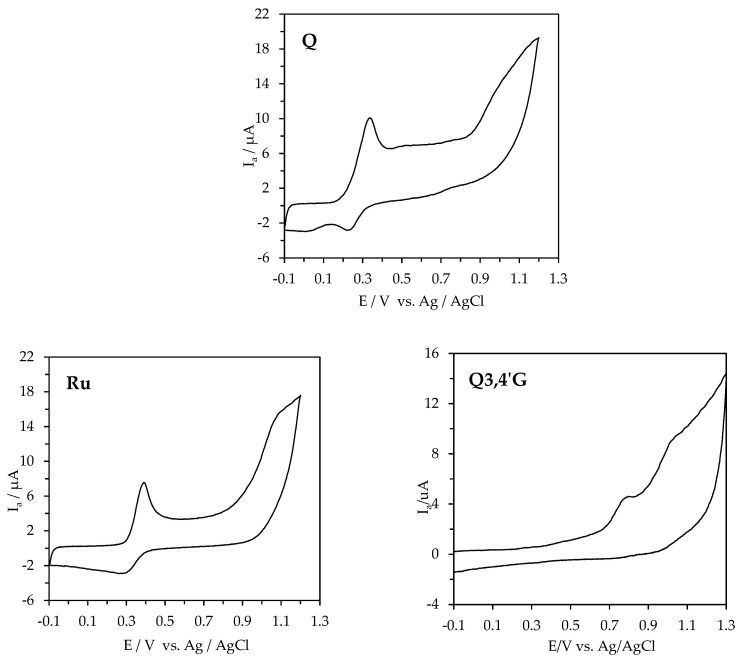

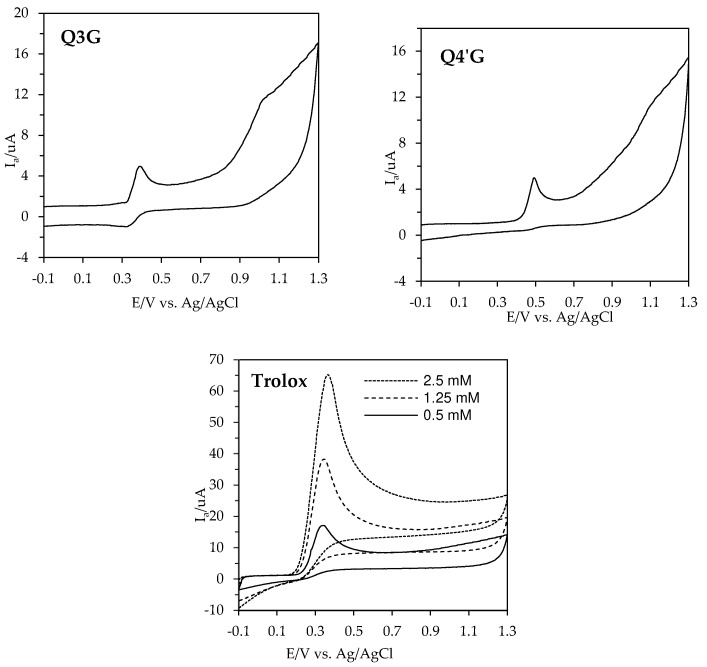
The cyclic voltammograms of 1 × 10^−4^ M of quercetin (Q), rutin (Ru), quercetin-3,4′-di-*O*-β-glucoside (Q3,4′G), quercetin-3-*O*-β-glucoside (Q3G), quercetin-4′-*O*-β-glucoside (Q4′G), and Trolox (0.5–2.5 mM) in 50 mM acetate–acetic buffer (pH 5.5) in 80% methanol; the scan rate was 100 mV s^−1^.

In this study, the antioxidant potentials of rutin, quercetin, and quercetin’s glucosides are described by their biological oxidation potential and their intensity of the anodic AC (Ia) in comparison to Trolox. To determine the antioxidant potential of these compounds the area under the AC wave was used, as was previously suggested [7,10,13]. Previously, the electrochemical behavior of quercetin has been studied; however, scant information is available about its glucosides and rutinoside [11,12]. The recorded cyclic voltammograms are shown in Figure 2.

The Q, Q3G, Ru, Q4′G, and Q3,4′G voltammograms showed well-defined oxidation waves with peak potentials of 310, 390, 410, 482, and 800 mV (vs. Ag/AgCl) in 50 mM acetate–acetic buffer (pH 5.5) in 80% methanol, respectively (Table 1).

In this study, the order of the antioxidant potential of rutin, quercetin, and quercetin’s glucosides was Q > Q3G > Ru ≈ Q4′G > Q3,4′G. This order reflects the current knowledge on the structural features and the nature of substitutions on rings B and C determining the antioxidant activity of flavonoids [47]. It is well recognized that the structural features and nature of substitutions on rings B and C determine the antioxidant activity of flavonoids [48,49]. It was also reported that the substitution of 3-OH subsequently reduced antioxidant activity, whilst the substitution of hydroxyl groups in ring B by methoxyl groups affected the free radical scavenging activity of flavonoids [50]. Our data indicates the importance of the 3′,4′-dihydroxy substitute on the B-ring combined with a 3-OH in ring C of quercetin and its glucosides for their electrochemical activity. Moreover, taking into account the values of the first oxidation potential of the studied compounds, Q can be described as a compound with a high antioxidant power (Ep < 0.3 V); Q3G, Ru, and Q4′G as compounds with intermediate potentials (0.3 V < Ep < 0.8 V); and Q3,4′G can be seen to be a compound with a low antioxidant power (0.8 V < Ep < 1.3 V). This conclusion was drawn in accordance with the work by Blasco et al. [51], in which the differentiation of the antioxidant power of phenolic compounds was based on the values of their oxidation potentials. With this evidence, the question regarding the impact of the antioxidant potential of rutin, quercetin, and quercetin’s glucosides on their multifaceted biological activities can be addressed.

### 2.2. Angiotensin-I-Converting Enzyme Inhibitory Activity of Quercetin and Its Glucosides

The ACE inhibitory activity of rutin, quercetin, and quercetin’s glucosides is shown in Table 2. The IC_50_ values for angiotensin-I-converting enzyme inhibition ranged from 59.3 µM to 143.5 µM in comparison to glutathione (IC_50_ = 41.7 µM) and captopril (IC_50_ = 0.0059 µM).

The IC_50_ value represents the concentration of each compound that inhibits ACE activity by 50%. The order of ACE inhibitory activity was Q > Q3,4′G > Ru > Q3G > Q4′G, thus indicating the importance of the structure–activity relationship. Our findings follow the study performed by Guerrero et al. [30]. They evaluated the in vitro ability of 17 flavonoids belonging to five structural subtypes to inhibit ACE in order to establish the structural basis of their bioactivity. The high ACE inhibitory activities, provided by the same fluorimetric method as in our study, showed quercetin and rutin to have IC_50_ values of 43 and 64 µM, respectively. Moreover, the higher ACE inhibitory activity of quercetin in comparison to Ru was also described by Tsai et al. [28], whereas the quercetin glucosides’ ACE inhibitory activities are shown for the first time in this study. These IC_50_ values of the quercetin glucosides were twice as high as the ACE inhibitory activity of quercetin, thus indicating their lower ACE inhibitory activity due to the substitution of hydroxyl groups by glucose in the catechol group in the B-ring and hydroxyl group at C3 in the C-ring. These findings follow other observations noting that the position and number of substituents in the basic flavonoid structure significantly affect the multifunctional activities of such molecules [52,53]. It is well known that antioxidant potential strongly depends on chemical structure, specifically on the number and position of hydroxyl groups, which may be the reason for their increased inhibitory effect on ACE activity. This relationship was previously described for the major green catechins [54] and hydroxybenzoic acids, differing only in terms of the number of hydroxyl groups on the benzene ring. Moreover, the implementation of in silico analysis or experiments involving cell lines to evaluate ACE inhibitory activity, as well as an analysis of potential mechanistic links from the perspective of the enzymes’ active sites, is also useful for the study of the ACE inhibitory activity of polyphenols [53].

Recently, a lot of bioactive compounds with potent ACE inhibitory activity have been separated and characterized from enzymatic hydrolysates of many foodstuffs [23,24,25,26,27,28,29,55,56]. The ACE inhibitory activity of flavonoids isolated from plants has been reported previously [57,58]. Both phenolic acids and flavonoids are able to modulate cardiovascular function by the regulation of blood pressure [59], oxidative stress [60], and inflammation [61]; however, their antihypertension activity has scarcely been investigated in relation to their antioxidant potential. In this study, a negative correlation was found between the IC_50_ values of rutin, quercetin, and quercetin’s glucosides and their antioxidant potentials provided by the cyclic voltammetry. The correlation coefficient had a value of r = −0.68, thus clearly indicating the impact of antioxidant potential and chemical structure on the ACE inhibitory activity of Q, Q3G, Q4′G, Q3,4′G, and Ru.

It can be concluded that the higher the antioxidant potential of the compounds, the higher the observed ACE inhibitory activity. However, the ACE inhibitory activity of rutin, quercetin, and quercetin’s glucosides was generally low as compared to captopril, a drug that treats high blood pressure, heart failure, and heart damage after a heart attack. On the other hand, the provided ACE inhibitory activities were comparable with that of GSH, thus indicating the importance of sources of glutathione, rutin, quercetin, and quercetin’s glucosides in treatment for increased blood pressure by dietary intervention or enhanced drug therapies.

### 2.3. Acetylcholinesterase (AChE) Inhibitory Activity of Rutin, Quercetin, and Quercetin’s Glucosides

Acetylcholinesterase (AChE) inhibitors are used for the treatment of Alzheimer’s disease (AD), increasing neurotransmitter acetylcholine levels at cerebral cortex synapses. In this study, we investigated the activity of rutin, quercetin, and quercetin’s glucosides to act against acetylcholinesterase (AChE) inhibition, and the data are shown in Table 3.

During the last decade, alkylpyridium polymers, dehydroevodiamine (DHED), and carbamate-type AChE inhibitors have been reported, but because of their bioavailability problems and possible side effects, there is still great interest in finding better AChE inhibitors [37,62].

In this paper, the acetylcholinesterase inhibitory activities of rutin, quercetin, and quercetin’s glucosides are provided for the first time. Galanthamine, licensed in Europe for AD treatment, was used due to its AChE activity inhibition [18,63,64]. Our IC_50_ values for AChE inhibition ranged from 1.331 µM to 2.496 µM in comparison to galanthamine (IC_50_ = 0.043 µM). Quercetin, its glucosides, and rutin showed the highest AChE inhibitory activity however their IC_50_ values were about 32–40 times higher compared to the activity of galanthamine. The highest IC_50_ value was noted for the Q3,4′G structure, and this value was statistically significantly different as compared to the remaining compounds. The order of the AChE enzyme inhibitory activity was galanthamine > Q ≈ Q3G ≈ Q4′G ≈ Ru > Q3,4′G, thus indicating a weak relationship with chemical structure.

As Q was defined as a compound with a high antioxidant potential; Q3G, Ru, and Q4′G as compounds with intermediate potential; and Q3,4′G as a compound with a low antioxidant power, their correlation with acetylcholinesterase inhibitory activity was not investigated. In this study, a negative correlation was found between the IC_50_ values of rutin (Ru), quercetin (Q), and quercetin’s glucosides and their antioxidant potentials as provided by cyclic voltammetry. The correlation coefficient had a value of r = −0.77, thus clearly indicating the impact of the antioxidant potential of Q, Q3G, Q4′G, Q3,4′G, and Ru on their AChE inhibitory activity.

### 2.4. The Inhibitory Activity of Rutin, Quercetin, and Its Glucosides Against Advanced Glycation Endproduct (AGE) Formation

During the last few decades, dietary flavonoids have been reported as effective AGE inhibitors contributing to the prevention of aging and diabetes complications [45,65]. The inhibitory activities of rutin, quercetin, and quercetin’s glucosides against AGE formation are shown in Table 4.

The IC_50_ values against AGE formation in the BSA/glucose model system ranged from 0.047 to 0.259 mM as compared to aminoquanidine (IC_50_ = 0.432 mM). The ranking of anti-AGE activity in the BSA/glucose model system was Q3G > Q3,4′G > Ru ≈ Q ≈ Q4′G > AG. The anti-AGE activity of Q3G was about twice as high in comparison to Q3,4′G; about four times higher in relation to Ru, Q, and Q4′G; and, finally, about nine times higher in relation to AG. Similarly, the IC_50_ values against AGE formation in the BSA/MGO model system were noted to range from 0.324 to 0.466 mM in relation to the IC_50_ of AG (0.531 mM). The ranking of anti-AGE activity in the BSA/MGO model system was Q3,4′G ≈ Q4′G ≈ Q3G > Ru ≈ Q > AG. The anti-AGE activities of Q3,4′G, Q4′G, and Q3G were higher by 27, 11, and 6% in comparison to Ru and finally higher by 64, 44, and 37% in relation to AG.

In this study, a positive correlation was found between the IC_50_ values of rutin (Ru), quercetin (Q), and quercetin’s glucosides obtained in the BSA/glucose and BSA/MGO model systems and their antioxidant potentials provided by cyclic voltammetry. The correlation coefficients had values of r = 0.37 (BSA/glucose) and r = 0.93 (BSA/MGO), thus clearly indicating the influence of the antioxidant potential of Q, Q3G, Q4′G, Q3,4′G, and Ru on the inhibition of AGE formation, especially in BSA/MGO system. These results indicate that quercetin glucosides display strong inhibitory capacity on AGE formation as compared to quercetin and aminoguanidine. Moreover, the IC_50_ values of rutin, quercetin, and quercetin’s glucosides obtained in the BSA/MGO model system were negatively correlated with their IC_50_ values for ACE inhibition (r = −0.67) and for AChE inhibition (r = −0.81), whereas no correlation was found between their ACE and AChE inhibition activities.

## 3. Materials and Methods

### 3.1. Chemicals

Rutin (quercetin-3-*O*-rutinoside), 6-hydroxy-2,5,7,8-tetramethylchroman-2-carboxylic acid (Trolox), sodium azide, bovine serum albumin (BSA), D-glucose, methyl glyoxal (MGO), aminoguanidine hydrochloride, angiotensin-converting enzymes (ACEs) from porcine kidneys (EC 3.4.15.1), and captopril were purchased from Sigma (Sigma Chemical Company, Saint Louis, MO, USA). Quercetin (Q) and quercetin-4′-*O*-β-glucoside (Q4′G) were acquired from Extrasynthese (Genay, France), quercetin-3-*O*-β-glucoside (Q3G) was acquired from Fluka, and quercetin-3,4′-di-*O*-β-glucoside (Q34′G) was acquired from Polyphenols Laboratories AS (4327 Sandnes, Norway). The substrate *o*-aminobenzoylglycyl-*p*-nitorphenylalanylproline (Abz-Gly-Phe(NO_2_)-Pro) was obtained from BACHEM (Bubendorf, Switzerland). Acetylthiocholine iodide (ATCI), acetylcholinesterase (AChE-type VI-S, from *Electrophorus electricus*), 5,5′[2-nitrobenzoic acid] (DTNB), and galanthamine hydrobromide were obtained from Sigma Chemical Co. (Poznań, Poland). Methanol, acetic acid (supra-gradient), and sodium acetate were acquired from Merck KGaA, Darmstadt, Germany. All the other reagents of reagent-grade quality were obtained from POCh, Gliwice, Poland. Water was purified with a Mili-Q system (Millipore, Bedford, MA, USA).

### 3.2. The Measurement of the Antioxidant Potency of Rutin, Quercetin, and Quercetin’s Glucosides in Voltammetric Experiments

A galvanostat G 750 (Gamry Ins., Warminster, PA, USA) with a conventional three-electrode system was used for the cyclic voltammetric experiments, with Ru, Q, Ru, Q4′G, Q3G, and Q34′G (0.1 mM in 80% methanol) or Trolox solutions (0.1–2.5 mM) mixed with 0.2 M sodium acetate–acetic buffer (pH 4.5 in 80% methanol) at a ratio of 1:1 (*v*/*v*), as has previously been described in detail [7,66]. The cyclic voltammograms were acquired in the range of −100 to 1300 mV at a scanning rate of 100 mV s^−1^. The linear response of a given Trolox concentration (y = 121.13x + 4.25; R^2^ = 0.99) was used to express the antioxidant activity of rutin, quercetin, and quercetin’s glucosides as mM of Trolox (n = 6).

### 3.3. Angiotensin-I-Converting Enzyme Inhibitory Assay

Standards of rutin, quercetin, and quercetin’s glucosides were dissolved in 80% methanol. Each standard was diluted to various concentrations using deionized water to determine its ACE inhibitory activity, expressed as an IC_50_ value. The ACE stock solution was prepared by dissolving enzyme in glycerol at 50% with Buffer A (protein concentration approximately 150 μg/mL), whereas the working solution was prepared by the dilution of the stock solution with Buffer B to make a protein concentration of approximately 7.5 μg/mL. The substrate stock solution was prepared by dissolving the whole content (50 mg) of Abz-Gly-Phe (NO_2_)-Pro (Bachem) in 10.33 mL of Buffer C (concentration approximately 10 mM), while the working solution was prepared by the dilution of the substrate stock solution with Buffer C to make a final Abz-Gly-Phe (NO_2_)-Pro concentration of approximately 0.45 mM. A captopril solution of 0.1 μM was prepared in deionized water. The angiotensin-converting enzyme (ACE) inhibitory activity assay was conducted according to the method of Sentandreu and Toldra [67]. The fluorescence generated was measured using a Tecan Infinite M1000 PRO multiscan microplate fluorometer (TK Biotech, Warsaw, Poland). To calculate the % of inhibition of ACE, the following equation was used:

Relative ACE activity % = 100 − (ΔRFU_sample_ ∗ 100/ΔRFU_negative control_)

where ΔRFU = RFU_at time 30_ − RFU_at time 0_.

The IC_50_ value, indicating the sample concentration at a 50% inhibition of ACE activity, was determined using linear regression analysis of logarithmic plots. At least 3 replicates for each standard solution were conducted. Results were expressed as IC_50_ values.

### 3.4. Acetylcholinesterase Inhibitory Assay

The AChE inhibitory activity of the pure standard compounds was evaluated following the methodology adapted from Eldeen et al. [68]. Three buffers were prepared: Buffer A (50 mM Tris–HCl, pH 8.0), Buffer B (50 mM Tris–HCl, pH 8.0, containing 0.1% bovine serum albumin), and Buffer C (50 mM Tris–HCl, pH 8.0, containing 0.1 M NaCl and 0.02 M MgCl_2_·6H_2_O). In a 96-well microplate, the following components were combined: 25 μL of 15 mM acetylthiocholine iodide (ATCI) dissolved in water, 125 μL of 3 mM 5,5′-dithiobis(2-nitrobenzoic acid) (DTNB) prepared in Buffer C, 50 μL of Buffer B, and 25 μL of the test compound solution at concentrations ranging from 0.01 to 1 mg/mL. The samples were sequentially diluted in a small volume of DMSO and Buffer A (1:99; *v*/*v*). Galanthamine (0.5–50 μg/mL in water) served as the positive control, while water was used as the negative control. Initial absorbance readings at 405 nm were recorded every 45 s for a total of five measurements to establish baseline activity. Subsequently, 25 μL of AChE solution (0.2 U/mL in Buffer B) was added to each well, and absorbance was measured eight times at 45 s intervals to monitor enzymatic activity. All the measurements were conducted using a microplate reader (Tecan Infinite M1000 PRO, TK Biotech, Warsaw, Poland). Each assay was performed in triplicate to ensure reproducibility. The inhibition of AChE activity was calculated as follows:

$$AChE\;inhibition\;\left(\%\right)=100-(∆{Abs}_{sample}*100/{Abs}_{negative\;control})$$

where ΔAbs (Absorbance) = Abs_with enzyme after 360 s_ − Abs_without enzyme 225 s_.

The IC_50_, indicating the concentration of the compound at which AChE activity was inhibited by 50%, was determined through linear regression analysis, with coefficients of determination (R^2^) ranging from 0.805 to 0.999, based on triplicate measurements.

### 3.5. The Inhibition of the Formation of the Advanced Glycation Endproducts (AGEs)

The positive control aminoguanidine and standards of rutin, quercetin, and quercetin’s glucosides were dissolved in DMSO and then in a phosphate buffer [69]. The inhibitory activity against the formation of AGEs was analyzed in BSA/glucose and BSA/MGO model systems, as reported by Thornalley [70], and the fluorescent intensity was measured. The percentage of inhibition of AGEs was calculated from the obtained fluorescence of the test solution with or without inhibitors.

### 3.6. Statistical Analysis

Results were given as the average ± standard deviation (SD). One-way analysis of variance (ANOVA) was used for the analysis of significant differences in the multifaceted biological activities of quercetin and its glucosides (*p* < 0.05) (GraphPad Prism version 9 for Windows, GraphPad Software, San Diego, CA, USA). Correlation analysis was performed and the Pearson correlation coefficients were calculated.

## 4. Conclusions

In this study, rutin (Ru), quercetin (Q), and quercetin’s glucosides (Q3G, Q4′G, and Q3,4′G) showed multifaceted biological activities. Q and Ru showed high ACE and AChE inhibitory activity, whereas quercetin’s glucosides were strong inhibitors of AGE formation. The order of ACE inhibitory activity was Q > Q3,4′G > Ru > Q3G > Q4′G, thus indicating the importance structure–antioxidant activity relationship. The order of AChE enzyme inhibitory activity was Q ≈ Q3G ≈ Q4′G ≈ Ru > Q3,4′G, and the correlation between the antioxidant potential and AChE inhibitory activity (r = −0.77) indicated the impact of antioxidant potential. The quercetin glucosides displayed a strong inhibitory capacity on AGE formation, as the ranking of anti-AGE activity in the BSA/MGO model system was Q3,4′G ≈ Q4′G ≈ Q3G > Ru ≈ Q > AG. The data on the inhibition of the angiotensin-converting enzyme (ACE) activity, acetylcholinesterase (AChE) activity, and advanced glycation endproduct (AGE) formation are important regarding hypertension, Alzheimer-type dementia, and diabetic complication, respectively. These effects of rutin, quercetin, and quercetin’s glucosides expand our knowledge of the multifunctional activity of biologically active compounds of plant origin, which can be useful in diet therapy and supporting drugs’ actions.

## Figures and Tables

**Table 1:** The oxidation and antioxidant activity of quercetin (Q), quercetin-3-*O*-β-glucoside (Q3G), quercetin-4′-*O*-β-glucoside (Q4′G), quercetin-3,4′-di-*O*-β-glucoside (Q3,4′G), and rutin (Ru) provided by the cyclic voltammetric method (n = 6).

Compound	First Oxidation Potential (mV)	Antioxidant Activity (mM Trolox)
Q	310 ± 4 ^e^	3.92 ± 0.20 ^a^
Q3G	390 ± 4 ^d^	2.59 ± 0.04 ^b^
Q4′G	482 ± 3 ^b^	1.99 ± 0.09 ^c^
Q3,4′G	800 ± 7 ^a^	1.07 ± 0.05 ^d^
Ru	410 ± 5 ^c^	2.04 ± 0.07 ^c^

Data are expressed as means ± standard deviations (n = 6). The equation of the linear regression of Trolox concentrations (y = 121.13 x + 4.25; R^2^ = 0.99) was used for the calculation of the antioxidant potentials. The means in a column followed by different letters are significantly different (*p* ≤ 0.05).

**Table 2:** The IC_50_ of rutin (Ru), quercetin (Q), and quercetin’s glucosides for ACE inhibition (µM).

Compound	Equation of Linear Regression	IC_50_ (µM)
Q	y = 0.3831x + 27.285 R^2^ = 0.96	59.29 ± 0.48 ^e^
Q3G	y = 0.253x + 15.79 R^2^ = 0.96	135.22 ± 1.04 ^b^
Q4′G	y = 0.268x + 11.553 R^2^ = 0.94	143.46 ± 1.44 ^a^
Q3,4′G	y = 0.2403x + 22.457 R^2^ = 0.94	114.62 ± 1.25 ^d^
Ru	y = 0.3206x + 11.355 R^2^ = 0.98	120.54 ± 1.56 ^c^
Captopril	y = 5008.1x + 20.632 R^2^ =0.98	0.00586 ± 0.00004 ^g^
GSH	y = 0.1337x + 44.429 R^2^ =0.98	41.68 ± 3.45 ^f^

The inhibitory effect of Q, Q3G, Q4′G, Q3,4′G, and Ru in comparison to captopril and reduced glutathione (GSH) within the concentration range of 1.0–0.01 mM was determined. The data are expressed as means ± standard deviations (n = 6). The equation of linear regression was used for IC_50_ calculation. The means in a column followed by different letters are significantly different (*p* ≤ 0.05).

**Table 3:** The IC_50_ values of rutin (Ru), quercetin (Q), and quercetin’s glucosides for acetylcholinesterase (AChE) inhibition (µM).

Compound	Equation of Linear Regression	IC_50_ (µM)
Q	y = 32.48x + 5.370 R^2^ = 0.99	1.374 ± 0.077 ^b^
Q3G	y = 29.376x + 10.913 R^2^ = 0.98	1.331 ± 0.111 ^b^
Q4′G	y = 37.843x − 14.26 R^2^ = 0.99	1.698 ± 0.144 ^b^
Q3,4′G	y = 15.828x + 10.495 R^2^ = 0.99	2.496 ± 0.239 ^a^
Ru	y = 32.702x + 2.0619 R^2^ = 0.99	1.466 ± 0.182 ^b^
Galanthamine	y = 834.84x + 14.195 R^2^ = 0.99	0.043 ± 0.008 ^c^

The data are expressed as means ± standard deviations (n = 6). The equation of linear regression was used for IC_50_ calculation. The means in a column followed by different letters are significantly different (*p* ≤ 0.05).

**Table 4:** The inhibitory activities of rutin (Ru), quercetin (Q), and quercetin’s glucosides against AGE formation (IC_50_).

Compound	BSA/Glucose Model System		BSA/MGO Model System	
	Equation of Linear Regression	IC_50_ (mM)	Equation of Linear Regression	IC_50_ (mM)
Q	y = 0.0164x − 0.5768 R^2^ = 0.86	0.243 ± 0.012 ^bc^	y = 0.0141x − 0.2341 R^2^ = 0.99	0.466 ± 0.017 ^b^
Q3G	y =0.0179x − 0.848 R^2^ = 0.83	0.047 ± 0.003 ^d^	y = 0.0112x − 0.171 R^2^ = 0.93	0.389 ± 0.022 ^c^
Q4′G	Y =0.0156x − 0.5208 R^2^ = 0.94	0.259 ± 0.016 ^b^	y =0.0131x − 0.285 R^2^ = 0.99	0.370 ± 0.023 ^cd^
Q3,4′G	y = 0.0163x − 0.7256 R^2^ = 0.73	0.089 ± 0.005 ^d^	y =0.0109x − 0.221 R^2^ = 0.88	0.324 ± 0.019 ^d^
Ru	y = 0.0141x − 0.5076 R^2^ = 0.75	0.197 ± 0.008 ^c^	y = 0.0132x − 0.237 R^2^ = 0.96	0.413 ± 0.024 ^bc^
AG	Y =0.0167x − 0.4031 R^2^ =0.94	0.432 ± 0.038 ^a^	y= 0.0161x − 0.274 R^2^ = 0.99	0.531 ± 0.028 ^a^

The equation of linear regression was used for IC_50_ calculation. The means in a column followed by different letters are significantly different (*p* ≤ 0.05).

## Data Availability

The original contributions presented in this study are included in the article. Further inquiries can be directed to the corresponding author.
